# Variability of Sentinel Lymph Node Location in Patients with Trunk Melanoma

**DOI:** 10.3390/diagnostics13172790

**Published:** 2023-08-29

**Authors:** Florin Bobirca, Mihaela Leventer, Dragos Eugen Georgescu, Dan Andrei Dumitrescu, Cristina Alexandru, Dragos Serban, Liana Valeanu, Traian Pătrașcu, Anca Bobircă

**Affiliations:** 1 Surgery Department, Carol Davila University of Medicine and Pharmacy, 050474 Bucharest, Romania; 2 Surgery Department, Dr. Ion Cantacuzino Clinical Hospital, 011437 Bucharest, Romania; 3 Dr. Leventer Centre, 011216 Bucharest, Romania; 4 Internal Medicine and Rheumatology Department, Dr. Ion Cantacuzino Clinical Hospital, 011437 Bucharest, Romania; 5 Department of Anesthesiology and Intensive Care, University of Medicine and Pharmacy “Carol Davila”, 050474 Bucharest, Romania

**Keywords:** trunk melanoma, SLNB, surgical treatment, lymphatic drainage

## Abstract

(1) Background: Melanoma is one of the most aggressive types of neoplasia, and the management of this pathology requires a correct staging, as well as a personalized modern oncological treatment. The main objective of the study is to determine the variability of the lymphatic drainage for patients with melanomas located on the trunk and, secondarily, to determine the features of individuals who underwent sentinel lymph node biopsy (SLNB) depending on the exact location on the trunk. (2) Methods: This retrospective, observational, single-center study included 62 cases of trunk melanoma operated between July 2019 and March 2023, in which SLNB was performed and a total of 84 lymph nodes were excised. (3) Results: Patients had a median age of 54.5 (33–78) years, with 58.1% being male; the melanomas had a median Breslow index of 2.3 (0.5–12.5) mm. Approximately 64.3% of the cohort had melanoma on the upper part of the trunk (54 cases) and 35.7% had it on the lower part (30 cases). The type of anesthesia chosen was general anesthesia in 53 cases and spinal anesthesia in 9 cases (85.5% vs. 14.5%, *p* < 0.001). The number of sentinel lymph nodes excised was 54 for melanomas located on the upper part of the trunk (8 cervical and 46 axillary) and 30 sentinel lymph nodes for melanomas of the lower part of the trunk (16 at the axillary level and 14 at the inguinal level). Out of the 54 LNs identified in patients with melanoma on the upper part of the trunk, 13 were positive, with a total of 12 positive lymph nodes (LNs) from the axillar basin, and only one from the cervical region. Additionally, the incidence of patients with a minimum of two identified sentinel lymph nodes was 32.2%, with a total of seven having LN involvement in two basins, and only one of these cases showed positivity for malignancy. (4) Conclusions: SLNBs were more frequent in the axillary region overall, and had more positive SLNs. Moreover, melanoma on the upper part of the trunk had a higher rate of positive SLNs compared to the lower part. Tumors located on the lower part of the truck had more positive SLNs in the axillary region than in the inguinal one.

## 1. Introduction

Melanoma represents a global health problem, as it is one of the most aggressive types of tumors. The treatment of this pathology has made great progress in recent years, with new staging techniques and the appearance of targeted molecules for systemic treatments.

Although, from an epidemiological point of view, it represents only 1% of skin tumors, this neoplasia accounts for approximately 325,000 new cases and 57,000 deaths annually [1]. Exposure to UVA and UVB radiation should be emphasized as one of the most significant risk factors, since these variables promote cellular alterations that result in an uncontrolled proliferation of melanocytes [2]. Another important aspect is the genetic component, with between 5 and 10% of patients with melanomas reporting that they have relatives with the same pathology; it is known that the risk of developing the disease is two times higher for those who have relatives with melanomas. Moreover, individuals with pale skin, blond or light hair, blue eyes, immunosuppressive conditions, or with a high number of nevi are more susceptible to developing melanomas [3].

Applying sunscreen with an SPF of at least 30 or doing a complete body inspection repeatedly over time to monitor changes in skin and identify suspicious lesions are two ways to prevent this pathology.

### 1.1. SLNB Concept

A cutaneous melanoma is characterized by the local proliferation of malignant melanocytic cells, followed by the extension to the lymphatic system, the main site of melanoma metastasis. A category of patients can develop satellite metastases located less than 2 cm from the primary tumor and in-transit metastases located within 2 cm from the first nodal site of drainage [4].

The key difference between a localized and a systemic disease is the dissemination of melanocytic cells into the lymph nodes. Furthermore, the most important prognostic factor in terms of survival in melanomas is represented by lymph node involvement [5]. When the tumor expands, the sentinel lymph node serves as the first filter for cancerous cells. The first mention of this concept was in the 1960s regarding parotid neoplasms, but the international recognition and validation of this method came in 1992, when Morton and Cochran used the technique on a group of patients with melanomas [6]. Grace to SLNB technique, it is now feasible to assess individuals in whom metastases are not found clinically or through imaging, and to also identify whether the disease is localized or has progressed to the stage of micrometastases.

As the SLNB technique is the most important staging technique in cutaneous melanomas, the indications have been intensively studied; nowadays, they are established according to the guidelines from the National Comprehensive Cancer Network (NCCN).

For tumors with a Breslow index below 0.8 mm, SLNB is necessary if the histopathological and immunohistochemical examination reveals signs of increased aggressiveness of the tumor, such as ulceration or an increased mitotic index. On the other hand, when evaluating patients with small-thickness melanomas (<1 mm), a proper assessment of the risk factors associated with sentinel node positivity is conducted using an SLNB.

According to the NCCN guidelines, SLNBs are recommended for patients in stage IB or stage II [7,8,9].

The utility of a sentinel lymph node biopsy after wide local excision is controversial due to the possible interruption of the lymphatic drainage. However, some studies indicate that the correlation between preoperative and postoperative lymphoscintigraphy following wide excision and tissue rearrangement is between 89 and 95% [10,11,12]. It is recommended to excise the sentinel lymph node(s) simultaneously with the resection and oncological margins, particularly if rotation flaps or skin grafts were used for re-construction, since they can interfere with the accuracy of the SLNB result [13,14].

### 1.2. The Sentinel Lymph Node—A Histopathological Report

Formalin fixation, paraffin embedding, sequential sectioning with hematoxylin-eosin staining, and immunohistochemical analyses with S100, HMB45, and MART-1 for molecular analysis remain the standard method for analysis of the SLN [15,16].

The number of positive nodes, the amount of tumor tissue from the lymph node, the location (subcapsular or intraparenchymal), as well as the presence of the extracapsular tumoral extension, are all identified in the histological analysis of the biopsied lymph nodes [17].

The Rotterdam criteria are used to classify patients according to the size of the tumor deposits into three groups: <0.1 mm, 0.1–1 mm, and >1 mm in diameter. Patients with deposits greater than 1 mm in diameter have a higher probability of testing positive for other regional lymph nodes in addition to having a poor melanoma-specific survival (MSS) [18,19].

In more than 50% of cutaneous melanomas, the BRAF mutation activates the MAP kinase/ERK signaling pathway. In patients with localized or widespread metastatic disease, examination is required for therapeutic decisions. The guidelines recommend retesting from the metastatic tissue as often as possible in patients with regional or systemic metastatic disease [20,21,22]. According to Ascierto et al., BRAF contributes to the progression of melanomas by stimulating the secretion of VEGF and the vascularization of tumors, as well as the factors that influence cell migration, integrin signaling, and cell contractility [22,23,24].

## 2. Materials and Methods

### 2.1. Study Design

This is an observational, retrospective, single-center study performed in a private clinic in Bucharest, Romania. A total of 62 cases of melanomas located on the trunk were included in this study; these were patients who underwent surgery between July 2019 and March 2023. The inclusion criteria were cases of melanomas on the trunk with a Breslow index of at least 0.8 mm and the absence of imaging and clinical detectable metastases. All patients included were over 18 years old, and signed the informed consent form. SLNB was performed at a maximum of 6 weeks from the melanoma biopsy. In our clinic, the indication to perform SLNB is obtained following a multidisciplinary meeting attended by doctors from the specialties of general surgery, plastic surgery, medical oncology, histopathology, dermatology, nuclear medicine, and the anesthesiologist, the team that analyses the cases and establishes the suitability for surgery. As many times as possible, surgery is performed on the same day with the injection of the radiotracer, so that the charge of the lymph node(s) would be as high as can be.

The exclusion criteria were: melanomas with distant metastases, patients with severe associated cardiovascular diseases or low performance indices that are contraindications for the anesthesia, age below 18 years, and melanoma patients with a location other than the trunk.

A total of 62 cases were included in the study, and further divided into two subgroups for the comparison analysis, as follows: the melanoma on the upper part of the trunk (UpM) represented by the anterior and posterior thoracic region, and melanoma of the lower part of the trunk (LoM), represented by the abdomen and the lumbar region.

### 2.2. SLNB Surgical Technique

The SLNB technique begins by performing a lymphoscintigraphy on the day of surgery (or, at the earliest, 24 h before it) when a radiotracer is injected (Technetium Tc-99m) at 1 cm distance from the scar of the excised melanoma. The nuclear medicine department provides the result of this investigation, highlighting the lymphatic route and the drainage to the sentinel lymph node(s). In order to increase the sensitivity of the detection, a blue dye is injected around the scar 15–20 min before surgery, in order to reach the sentinel lymph node(s). The area where the sentinel lymph node is located will be checked with the gamma probe, and the incision will be made to provide the best possible surgical exposure and also to facilitate the dissection of the surrounding structures. Finally, the identification of the sentinel lymph node is done using a dual method: with the gamma probe, thus determining the radioactivity of the lymph node, and the recognition of coloring from the blue dye.

It should be mentioned that the measurement of the lymph node radioactivity is done in three moments: preoperative, intraoperative and ex vivo. After the sentinel lymph node(s) has been excised, the operating field will be double-checked for the possibility of detection of another radioactively charged lymph node (if it has a radioactive value of more than 10% of the value from the “hottest lymph node”).

### 2.3. Data Collection

The data collected were demographic, such as age, sex, and BMI, and also related to the melanoma, such as tumor stage, Breslow index, location of sentinel lymph nodes, and node positivity. Surgery characteristics were registered as well, like type of anesthesia and average time of surgery.

### 2.4. Statistical Analyses

The results were reported as number and frequency for ordinal values and median with minimum and maximum value for numerical data. The analysis was performed using SPSS version 20.0. The tests implemented for the comparison were Pearson’s chi square test and Mann–Whitney U test, with a significant result at *p* < 0.05.

## 3. Results

In this study, a total of 62 cases were analyzed. Among them, 62.9% (39 cases) were diagnosed with melanoma on the upper part of the trunk, while the remaining 37.1% (23 cases) were diagnosed with melanoma on the lower part of the trunk. The characteristics of patients, and the comparisons between melanomas on the upper and lower part of the trunk cases, are shown in Table 1. The median age of the patients included in the study was 55 years, with a range of 34 to 78 years, and patients diagnosed with LoM had a median age of 53 years (min-max = 33–77). In terms of gender distribution, 36 cases (58.1%) out of the total were male, with 22 cases (56.4%) among the LoM group being male. Additionally, the study examined the body mass index (BMI) of the patients, which showed a median of 23.7 kg/m^2^ (ranging from 19.8 to 30.6 kg/m^2^) for UpM cases and 24.1 kg/m^2^ (ranging from 19.9 to 29.2 kg/m^2^) for LoM cases, indicating no significant difference between the two groups (*p* = 0.291). The tumor stage distribution revealed that, among all cases, 32.3% were classified as pT1, 14.5% as pT2, 24.2% as pT3, and 29.0% as pT4. When comparing UpM and LoM, no statistically significant differences were observed in the distribution of tumor stages (*p* = 0.413).

When comparing UpM with LoM groups regarding the Breslow index, there was no significant difference (median of 2.3 (0.5–12.5) for UpM vs. 2.3 (0.8–9.3) for LoM, *p* = 0.511).

Regarding the number of LN excided per patients, 44 patients had only one LN biopsy, while 18 had two LNs excised, and there were two cases with three LNs.

The primary lymph node localization was examined, and the analysis revealed that 9.7% of cases had cervical LN involvement, 71.0% had axillary LN involvement, and 19.4% had inguinal LN involvement. When comparing upper and lower melanomas, a statistically significant difference was observed in the distribution of LN localization (*p* < 0.001), with UpM cases showing a higher percentage of axillary LN involvement (84.6%) compared to LoM cases (33.3%).

Regarding the secondary LN localization, a subgroup analysis was conducted on 20 cases. Among them, 10.0% had cervical LN involvement, 80.0% had axillary LN involvement, and 10.0% had inguinal LN involvement. Furthermore, analyzing the LN involvement for the LoM group, for the first LN, almost half of the patients had axillary involvement (47.8%) while, for the second LN excided, 66.7% were from inguinal region, with an additional third lymph node from inguinal site as well.

The presence of a positive LN was investigated, and it was observed that 25.8% of all cases had positive LN involvement, with 30.8% in UpM patients and 17.4% in LoM patients (*p* = 0.245).

The type of anesthesia administered during surgery was also analyzed. Among patients with UpM, all cases underwent general anesthesia, while none received local anesthesia. In contrast, patients with LoM had 39.1% of cases which only required spinal anesthesia, with a statistically significant difference between the two groups (*p* < 0.001).

The surgery duration had a median of 120.0 min (ranging from 75 to 195) for UpM cases and 110.0 min (ranging from 70 to 200) for LoM cases. Although the difference was not statistically significant (*p* = 0.056), it suggests a slightly longer surgery duration for UpM cases.

Regarding the postoperative outcomes, the rate of reintervention was low, with only 1.6% of all cases requiring additional surgery. The only case which required reintervention belonged to LoM group (2.6%).

Additionally, the presence of the BRAF gene was registered in a subset of 14 cases, with a total of 10 cases having a positive BRAF gene. Among them, six were from patients with UpM and four from patients with LoM, with no significant difference between the two groups (*p* =1.000). This indicates a similar prevalence of the BRAF gene in both upper and lower trunk melanoma.

The total number of sentinel lymph nodes identified in the study was 84, the distribution is illustrated in Table 2. Among these, 54 (64.3%) were from patients diagnosed with UpM, with 13 (24.1%) of them being positive for malignancy. In contrast, 30 (35.7%) were associated with LoM and, out of these, 6 (20%) were positive for malignancy (*p* = 0.264).

In terms of lymph node involvement, the analysis revealed that, among all cases of trunk melanoma in this study, 9.5% had cervical LN involvement, 73.8% had axillary LN involvement, and 16.7% had inguinal LN involvement.

**Figure 1:**
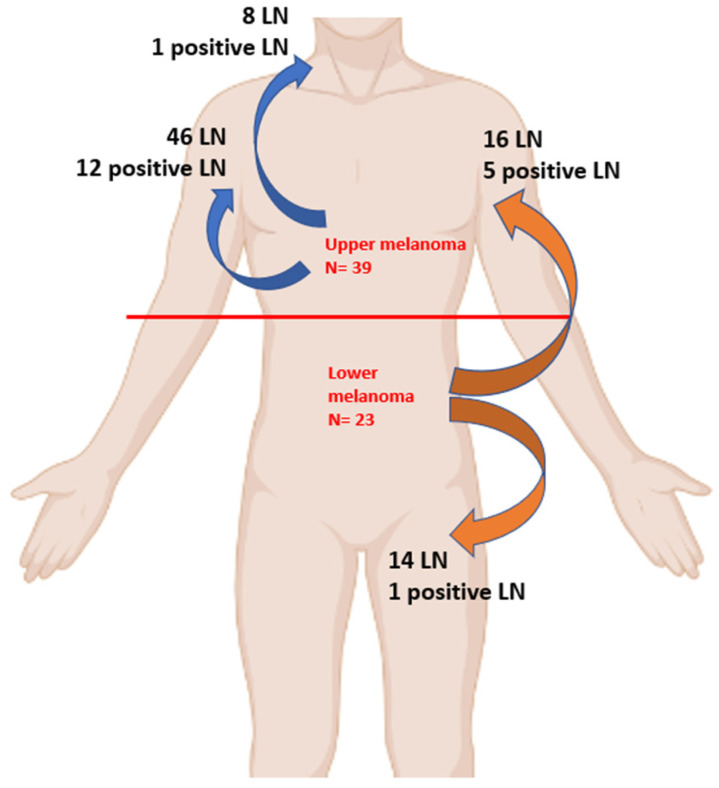
Graphical representation of the lymph node drainage.

As illustrated in Figure 1, in melanoma on the upper part of the trunk, out of the 54 LN patients identified, 13 were positive, with a total of 12 positive LN from axillar basin and only one from cervical region.

On the other hand, for cases diagnosed with melanoma on the lower part of the trunk, no LN involvement was observed in the cervical LN. However, 16 of cases out of 30 had axillary LN involvement. From the LoM subgroup, a total of six cases had positive LN, with five cases in axillar region and only one in inguinal region.

Moreover, analyzing the variability of multiple basin drainage, among the 20 patients with a minimum of two sentinel lymph nodes identified, seven had LN involvement in two basins. However, only one of these cases showed positivity for malignancy, specifically in the posterior thoracic site with axillary and cervical drainage.

Furthermore, among the 20 patients with a minimum of two sentinel lymph nodes, 15 had UpM (with five cases showing drainage in two basins), while five had LoM (with two cases showing drainage in two basins).

## 4. Discussion

This study highlighted the distribution of lymph nodes regarding the melanoma location on trunk. From a total of 62 patients, melanoma on the upper part of the trunk had a higher frequency than those on the lower part (62.9% vs. 37.1%). The median age of the patients included in the study was 54.5 years for the whole group, and 55 years for patients diagnosed with UpM; these results are comparable to that obtained by a Dutch study carried out on a larger group of 1192 patients with melanomas located in the upper trunk, where the average age obtained was 56 years old [25].

Related to the gender distribution, the study emphasized the fact that the male gender predominates, with 58.1% of cases; this value being close to that obtained by an American trial based on a cohort of 178,000 cases from the period 2004–2014 where, in the case of melanomas located on the trunk, 66.1% were male patients [26]. Moreover, in our study, male gender was more frequent among subjects diagnosed with melanoma on the lower trunk than those with the upper location. Another study carried out in Sweden shows a predominance of the male sex in the case of melanomas of the trunk, the proportion being 53%, compared to 47% for the female sex [27].

Another important aspect of the current study is represented by the Breslow index, the median value being 2.3 mm for both studied trunk regions. The specialized literature confirms this value, a study conducted in the USA that included 818 cases of melanoma of the trunk with an average Breslow index value of 2.3 mm [28].

However, the distribution according to the T stage of the tumor differs a lot from other populations. In the current work, T1 tumors represented the highest percentage of cases, 32.3%, while T2 represented the other extreme, 14.5% of cases; the literature shows completely different values, with tumors in stages T3 and T4 representing most of the cases, at 35% and 22%, respectively [27].

A study carried out in Portugal shows a different distribution regarding the T staging of tumors, with the exception of T3, where the values are almost equal. This study also shows a predominance of tumors located at the level of the upper trunk, this result being in accordance with what we obtained in our study, with the tumors located at the upper level being 62.9% [29].

Regarding the identification of the sentinel lymph node, the results of the study showed that there are tumors that can drain in one lymphatic way, others through two ways, and some of them can even have three sentinel lymph nodes. In the specialized literature, it is confirmed that melanomas located on the trunk are those that could have multiple lymphatic drainage regarding the sentinel lymph node; a study conducted on 135 patients with 61 cases of multiple drainage (45.2%) [29], where another study showing that in 352 trunk melanomas, 77 cases had multiple drainage (21.9%) [30]. It should be mentioned that another study carried out in Italy on 656 patients revealed that 167 cases had multiple drainage (25.4%) [31]. All these results are comparable to those obtained in our study, in which there are 20 patients with multiple drainage, comprising 32.2% of the cases.

The variability of the lymphatic channels is given by the location of the primary trunk tumor in relation to the cervical, axillary, and inguinal lymphatic basins, with significant statistical results when compared UpM and LoM in our findings (*p* < 0.05). Patients in the UpM subgroup had predominantly sentinel lymph nodes in the axillary lymph basin (84.6% for the first LNB, 85.7% for the second LNB, and 100% for the third LNB, respectively). In contrast, for LoM, the results of LN location varied if the first sentinel node was in somewhat similar proportions between the axillary region and groin (47.8% vs. 52.2%); in the case of the second sentinel lymph node, the axillary region had two thirds of the cases (66.7% vs. 33.3%). In both regions, both melanomas on the upper and lower parts of the trunk, there was one case with a third sentinel lymph node located in the axilla.

The positivity of the sentinel lymph node is closely related to the T stage of the melanoma and to its Breslow index. In our study, which had a total of 62 patients, 84 lymph nodes were analyzed, with 22.6% being positive. In a comparative study in which there were 39 patients with melanomas and in which SLNB was performed, a total of 121 lymph nodes were excised, of which 22 positive nodes (18.1%) were found at the histopathological examination, totaling 13 patients with a positive sentinel node [25].

Regarding the duration of the surgical intervention, there is an important difference between the two locations, i.e., the superior and inferior location of trunk melanoma. It should be mentioned that this duration involves a sentinel lymph node biopsy, an excision within oncological safety margins, and the closure of the wound; in some areas, a graft taken by the plastic surgeon is needed. The median time spent more with interventions on the upper trunk presupposes dissection in areas with lymphatic basins and important vascular structures, as we find in the cervical lymph nodes area [28].

The type of anesthesia chosen represents another issue in these interventions. At the level of the lower trunk, if we can opt for a spinal anesthesia that can reach both the area of the melanoma and the region of the sentinel lymph node, for melanomas located at the level of the upper trunk, general anesthesia was preferred in its entirety by oro-tracheal intubation, with the difference between the two being statistically significant (*p* < 0.001).

Another important feature of our study is represented by melanomas on the lower part of the trunk that had sentinel nodes located at the axillary level, with 16 out of 30 nodes (53.3%) being present in this lymphatic basin. Also, another interesting result obtained is that among the positive sentinel nodes of melanomas of the lower trunk, five out of six nodes (83.3%) are at the axillary level, something that could be further investigated on a larger cohort in order to research the lymphatic pathways of melanomas depending on the location of the primary tumor. In the literature, another study that highlighted lymphatic drainage in different locations of melanomas showed that, in the case of 67 patients with trunk melanomas who underwent SLNB, 35 axillary nodes (28.6%) were detected [32]. The presence of a positive LN varied between the groups, with a higher percentage observed in UpM cases, with the axillar basin being the most frequent location for all trunk melanomas (85.1%).

In our opinion, there are three main limitations in our study. First the small number of patients, making it necessary to develop further studies with larger cohorts. Second, the single center characteristic of the study, as we performed our study in a private unit, since there are only a few centers for SLNB in Romania. And third, data are lacking regarding the subtype of melanoma, information which could bring valuable results.

## 5. Conclusions

These findings contribute to our understanding of the characteristics and outcomes of melanomas located on the trunk, focusing on the comparison based on the affected area. The most frequent lymphatic drainage site for melanomas on the trunk was the axillary one, compared to cervical and inguinal. Moreover, the rate of positive SLNBs was higher for the axillary region, even in cases for melanomas located on the lower part of the trunk, emphasizing the importance of this region in terms of lymph node biopsy.

## Figures and Tables

**Table 1:** Characteristics of trunk melanoma patients and comparison between upper and lower melanoma trunk.

	Total N = 62	Upper Trunk Melanoma 39, (62.9%)	Lower Trunk Melanoma N = 23, (37.1%)	*p*-Value
Age (years) median (min-max)	54.5 (33–78)	55 (34–78)	53 (33–77)	0.782
Sex Masculine n,%	36, (58.1%)	22, (56.4%)	14, (60.9%)	0.731
BMI median (min-max)	23.9(19.8–30.6)	23.7 (19.8–30.6)	24.1 (19.9–29.2)	0.291
Tumor stage n,%				0.413
pT1	20, (32.3%)	14, (35.9%)	6, (26.1%)	
pT2	9, (14.5%)	4, (10.3%)	5, (21.7%)	
pT3	15, (24.2%)	11, (28.2%)	4, (17.4%)	
pT4	18, (29.0%)	10, (25.6%)	8, (34.8%)	
Breslow mm median (min-max)	2.3 (0.5–12.5)	2.3 (0.5–12.5)	2.3 (0.8–9.3)	0.511
1o LN localization n,%				<0.001
Cervical	6, (9.7%)	6, (15.4%)	0	
Axillar	44, (71.0%)	33, (84.6%)	11, (47.8%)	
Inguinal	12, (19.4%)	0	12, (52.2%)	
2o LN localization n,%				0.057
N = 20				
Cervical	2, (10.0%)	2, (14.3%)	0	
Axillar	16, (80.0%)	12, (85.7%)	4, (66.7%)	
Inguinal	2, (10.0%)	0	2, (33.3%)	
3o LN localization n,%				-
N = 2				
Cervical	0	0	0	
Axillar	2, (100%)	1, (100%)	1, (100%)	
Inguinal	0	0	0	
Positive LN n,%	16, (25.8%)	12, (30.8%)	6, (17.4%)	0.245
Period to LNB (days) median (min-max)	29.0 (22.0–40.0)	29.0 (22.0–40.0)	28.0 (22.0–39.0)	0.373
BRAF gene, n,%	10, (71.4%)	6, (66.7%)	4, (80.0%)	1
N = 14				
Anesthesia type n,%				<0.001
General	53, (85.5%)	39, (100%)	14, (60.9%)	
Local	9, (14.5%)	0	9, (39.1%)	
Surgery duration (minutes) median(min-max)	115.0 (70.0–200.0)	120.0 (75.0–195.0)	110.0 (70.0–200.0)	0.056
Reintervention n,%	1, (1.6%)	1, (2.6%)	0	1
Period of healing melanoma site (days) median (min-max)	12.0 (10.0–21.0)	12.0 (10.0–21.0)	12.0 (10.0–18.0)	0.833
Period of healing LN (days) median (min-max)	8.0 (7.0–8.0)	8.0 (7.0–9.0)	8.0 (7.0–8.0)	0.373

Abbreviation: N = number, LN—lymph node, min = minimum, max = maximum, % = percentages.

**Table 2:** Lymph node distribution.

N = 84	Cervical LN 8, (9.5%)	Axillar LN 62, (73.8%)	Inguinal LN 14, (16.7%)
Upper Melanoma, N = 54, 64.3%	8, (100%)	46, (74.2%)	0
Positive	1	12	
Lower Melanoma, N = 30, 35.7%	0	16, (25.8%)	14, (100%)
Positive		5	1

Abbreviations: N = number, LN = lymph node.

## Data Availability

The data presented in this study are available in the article.
